# Biochemical characteristics associated with spironolactone use in hospitalized patients with hypotonic hyponatraemia

**DOI:** 10.1080/07853890.2026.2718576

**Published:** 2026-09-17

**Authors:** Josef Klhůfek, Martin Vodička, Martin Fajkus, Ilja Ryšavý, Vladimír Kojecký, Tomáš Šálek

**Affiliations:** a Department of Clinical Pharmacy, Tomas Bata Hospital Zlin, Zlín, Czech Republic; b Institute of Clinical Biochemistry and Diagnostics, Medical Faculty in Hradec Králové, Charles University in Prague, Prague, Czech Republic; c Faculty of Applied Informatics, Tomas Bata University in Zlín, Zlín, Czech Republic; d Department of Internal Medicine, Tomas Bata Hospital Zlin, Zlín, Czech Republic; e 2nd Department of Internal Medicine, St. Anne’s Faculty Hospital, Brno, Czech Republic; f Faculty of Medicine, Masaryk University, Brno, Czech Republic; g Department of Clinical Biochemistry and Pharmacology, Tomas Bata Hospital Zlin, Zlín, Czech Republic

**Keywords:** Spironolactone, hyponatremia, diuretic-induced hyponatremia, drug-induced hyponatremia, furosemide

## Abstract

**Background:**

Spironolactone has been associated with hypotonic hyponatraemia. However, its accompanying biochemical pattern – especially with concomitant loop diuretics – remains underdescribed. We characterized biochemical and epidemiological profiles of hospitalized patients with established hypotonic hyponatraemia according to spironolactone exposure.

**Methods:**

Single-centre retrospective cohort of non-thiazide patients admitted with hypotonic hyponatraemia. Biochemical parameters were compared between spironolactone users (*n*=107) and non-users (*n*=427). The spironolactone-alone subgroup (*n*=27) was compared between (i) the non-diuretic group (*n*=374) and (ii) the spironolactone+furosemide group (*n*=80). We performed correlations between (a) admission serum sodium (s-Na) and in-hospital mortality; (b) spironolactone dose and biochemical parameters, also adjusted for furosemide dose; (c) serum potassium (s-K) and relevant parameters. Multivariable regression analyses were performed for s-K, s-Na, fractional excretion (FE)-K, FE-Cl, FE-H_2_O and serum urea (s-urea) as dependent variables to assess associations with spironolactone while accounting for potential confounders.

**Results:**

After eGFR adjustment, spironolactone users showed higher s-K (*p*<0.001), s-urea (*p*<0.001) and s-uric acid (s-UA; *p*=0.01). The spironolactone-alone group showed higher s-K (*p*=0.003), and s-urea (*p*=0.001) compared to the non-diuretic group; lower dose (*p*=0.04), s-urea (*p*=0.04), serum creatinine (s-creatinine) (*p*<0.001), s-UA (*p*=0.02) and chronic heart failure prevalence (*p*<0.001); higher eGFR (*p*=0.002), and hypertension prevalence (*p*=0.048), compared to the spironolactone+furosemide group. Multivariable regression analyses identified independent associations of spironolactone with higher s-K (*b*=0.33, *p*=0.001) and with higher log-transformed s-urea (*b*=0.22, *p*<0.001), corresponding to a 24.5% increase in s-urea.

**Conclusions:**

Spironolactone was independently associated with higher s-K and with higher s-urea levels. This pattern was not reflected by reduced urinary potassium excretion. Importantly, no clear evidence that concomitant furosemide materially modified this biochemical pattern was observed in the present analyses. Awareness of this pattern may support the phenotypic characterization of hyponatraemic patients receiving spironolactone. However, prospective studies are warranted to evaluate the diagnostic utility of this biochemical pattern in hyponatraemic patients receiving spironolactone.

## GRAPHICAL ABSTRACT

**Figure:**
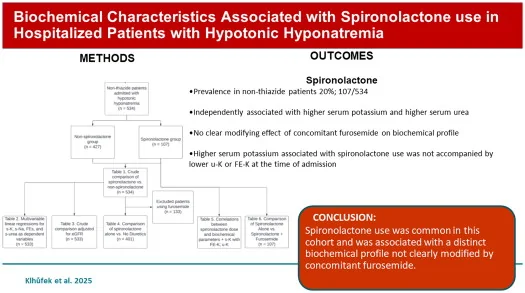

## 1. Introduction

Spironolactone, a synthetic steroidal antagonist of the mineralocorticoid receptor, was first synthesized in 1957 [1]. Since its introduction into clinical practice, it has become an essential therapeutic agent in the management of primary hyperaldosteronism, chronic heart failure and liver cirrhosis with associated ascites [2,3]. Moreover, spironolactone plays an established adjuvant role in the treatment of resistant hypertension [4]. Beyond its mineralocorticoid receptor antagonism, spironolactone exhibits significant antiandrogenic properties, which are clinically utilized in the management of acne vulgaris [5] and hirsutism in women [6], as well as in transgender individuals assigned male at birth [7].

A well-recognized and, in some cases, potentially life-threatening adverse effect of spironolactone therapy is hyperkalaemia. Other treatment-limiting adverse effects associated with spironolactone include gynaecomastia, hyponatraemia, metabolic acidosis, as well as gastrointestinal and neurological complications [2,3].

In the context of hyponatraemia, the underlying mechanism is linked to the direct on-target pharmacological action of spironolactone within the principal cells of the nephron collecting duct. Aldosterone receptor antagonism leads to reduced expression of epithelial sodium channel (ENaC) and the Na^+^/K^+^-ATPase, resulting in increased renal sodium excretion [8]. Further, this dose-dependent natriuretic effect of spironolactone [9] may lower effective arterial blood volume (EABV), thereby stimulating the non-osmotic release of antidiuretic hormone (ADH) [10].

In the context of other causes of hypotonic hyponatraemia, spironolactone is not typically considered among the most common etiologies. Moreover, the clinical conditions for which spironolactone is prescribed, such as heart failure and liver cirrhosis with associated ascites, are themselves well-established causes of hypotonic hyponatraemia and may potentially mask its hyponatraemia-inducing effect [11]. However, a fall in serum sodium (s-Na) during spironolactone therapy is well documented [12], including the associated risk of developing clinically significant hyponatraemia [13–16]. The strongest available evidence linking spironolactone use with hyponatraemia comes from a large retrospective population-based case-control study focused on hospitalization due to hyponatraemia [14]. After adjustment for confounding factors, spironolactone showed an adjusted odds ratio (aOR) of 1.96 (95% confidence interval [CI]: 1.76–2.18). For reference, thiazide diuretics – well-established culprits in hypotonic hyponatraemia – showed an aOR of 3.4 (95% CI: 3.2–3.7) in a similarly designed study [17]. Conversely, furosemide, which is frequently co-prescribed with spironolactone, has been associated with a reduced risk of hypotonic hyponatraemia [14].

Despite previously reported associations between spironolactone use and hyponatraemia, the epidemiological and biochemical characteristics of hyponatraemic patients receiving spironolactone remain insufficiently characterized in the literature. Furthermore, data reflecting the use of spironolactone in real-world clinical practice, where it is frequently prescribed in combination with other classes of diuretics, are scarce.

The present study aims to address this gap by characterizing the epidemiological and biochemical profiles associated with spironolactone use in hypotonic hyponatraemia. Primarily, this is achieved by comparing hyponatraemic patients receiving spironolactone with those not receiving spironolactone while accounting for potential confounders. Second, it further explores potential dose-dependent relationships between spironolactone use and biochemical parameters at hospital admission. Moreover, it examines the modifying role of concomitant furosemide therapy, which is frequently co-administered with spironolactone and is known to affect renal electrolyte handling.

## 2. Methods

### 2.1. Study design and dataset origin

This is a retrospective, single-centre cohort study analysing the non-thiazide arm of a previously published retrospective cohort of patients hospitalized with thiazide-associated hyponatraemia [18]. For the present analysis, we specifically selected patients not receiving thiazide diuretics in order to eliminate the well-established effect of thiazides on the biochemical profile of hyponatraemic patients. This approach allows a more accurate evaluation of spironolactone-associated biochemical patterns and reflects a real-world clinical perspective, in which spironolactone is often used in combination with loop diuretics and in patients with conditions that may themselves represent plausible etiologies of hyponatraemia.

### 2.2. Analytical framework

This study was exploratory in nature. The analyses focused on several clinically relevant domains: (i) biochemical differences between hyponatraemic patients receiving spironolactone and those not receiving spironolactone; (ii) the robustness of these differences after adjustment for clinically relevant covariates; (iii) the potential modifying role of concomitant furosemide therapy; (iv) spironolactone dose and biochemistry associations; and (v) the relationship between admission s-Na and in-hospital mortality.

### 2.3. Inclusion and exclusion criteria

We enrolled patients admitted to the Internal Medicine Department of the Tomas Bata Hospital Zlin between 1 January 2016 and 31 December 2022 with a diagnosis of hypotonic hyponatraemia (defined as s-Na ≤ 135 mmol/L and serum osmolality (s-osmolality) < 280 mmol/kg). Serum osmolality was calculated by the formula: s-osmolality = (2 × serum Na (s-Na) [mmol/L]) + serum urea (s-urea) [mmol/L] + serum glucose (s-glucose) [mmol/L]) and/or directly measured. *Exclusion criteria*: One of the measured or calculated s-osmolality ≥ 280 mmol/kg; the need for dialysis; age under 18 years; and thiazide use.

### 2.4. Patient enrolment

**Figure 1.**
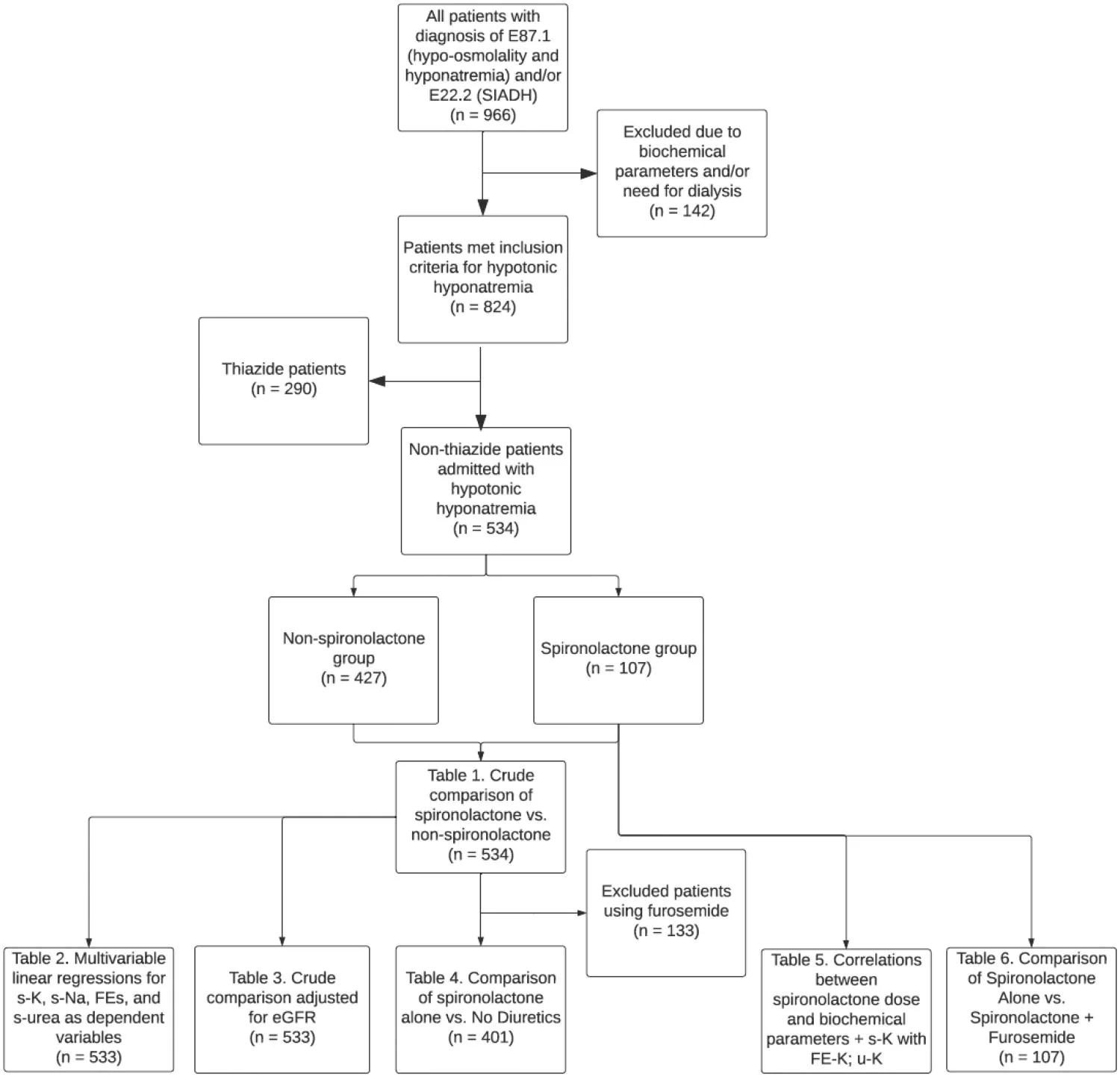
Study assembly.

Patients were identified by retrospective data analysis of the hospital’s digital registries. All patients were hospitalized in the Internal Medicine Department during the inclusion period for the diagnosis of E87.1 (hypo-osmolality and hyponatraemia) and/or E22.2 (syndrome of inappropriate antidiuretic hormone secretion; SIADH) according to the International Statistical Classification of Diseases and Related Health Problems, 10th revision (ICD-10). The study assembly is shown in Figure 1.

### 2.5. Data collection

Blood samples and urine spots were obtained at admission. The biochemical analysis of blood samples included s-Na, serum potassium (s-K), serum Cl (s-Cl), s-osmolality, s-urea, serum creatinine (s-creatinine), serum uric acid (s-UA) and s-glucose. Urine spots were biochemically analysed for urine osmolality (u-osmolality), urine Na (u-Na), urine K (u-K), urine Cl (u-Cl) and urine creatinine (u-creatinine). The estimated glomerular filtration rate (eGFR) was calculated by the CKD-EPI formula based on s-creatinine. Fractional excretions (FEs) of Na, K, Cl and uric acid (UA) were calculated by the formula FE = (u-Ion × s-creatinine)/(s-Ion × u-creatinine) × 100.

Clinical characteristics and medication history were recorded as part of the admission protocol or obtained from the patient during their hospital stay or from their caretakers if the patient was not conscious at admission. Mortality was obtained directly from the hospital’s digital register. All data were analysed retrospectively.

### 2.6. Statistical analysis

Normal distribution was tested using the Shapiro–Wilk test. Since almost all parameters are not normally distributed, we used non-parametric tests for further processing. Group comparisons of categorical variables were done with a chi-square test. For group comparisons of quantitative variables, the Mann–Whitney test for two medians was carried out. Correlations of quantitative variables were assessed using Spearman’s rank correlation. Statistical dependence of s-Na and in-hospital mortality was assessed using Fisher’s exact test. The values of biochemical parameters were adjusted for the eGFR value or furosemide dose. This covariate adjustment was performed using a residual-based method. For each parameter, a separate simple linear regression model was fitted in the pooled sample of patients with available data, with the parameter of interest as the dependent variable and the respective covariate as the independent variable. For each patient, the adjusted value was calculated by adding the individual regression residual, defined as the difference between the observed and model-predicted value, to the overall mean of the respective parameter. The resulting values therefore represent values standardized to the mean level of the covariate in the analysed sample. Multivariable linear regression analyses were performed using the enter approach, with all covariates considered clinically relevant entered simultaneously into the model. Normality of residuals in multiple linear regression was assessed using normal probability plots. S-urea, FE-Cl, FE-K and FE-H_2_O exhibited a right-skewed distribution and were log-transformed to improve symmetry and stabilize variance. In contrast, s-Na and s-K followed a near-normal distribution within physiological ranges and were analysed in their original scale, facilitating their direct clinical interpretability. Multicollinearity was assessed using the variance inflation factor (VIF) evaluation. A *p* value < 0.05 was considered statistically significant. Missing data were handled using a complete-case approach; no imputation was performed. The number of available observations for each variable is reported in the respective tables. The statistical analyses were performed using MedCalc Statistical Software, Version 23.0.6 (MedCalc Software Ltd., Ostend, Belgium; 2025) and NCSS 2026, Version 26.0.3 (NCSS, LLC, Kaysville, UT).

### 2.7. Ethics approval

Written informed consent was obtained from all the patients as a part of the informed consent to hospital admission. The informed consent included permission for the publication of anonymized hospitalization-related data for scientific purposes. The study was conducted in accordance with the ethical principles of the Declaration of Helsinki and was approved by the Ethics Committee of The Tomas Bata Hospital (No. 2024-34).

## 3. Results

Five hundred and thirty-four patients met the inclusion criteria for the analysis of the non-thiazide arm. The number of patients included in each analysis varied according to data availability and is specified in the corresponding tables. The prevalence of spironolactone was 20.0% (107/534). In-hospital mortality was not associated with s-Na at admission (*p* = 0.23).

A crude comparison of demographical, biochemical and clinical characteristics of the spironolactone (*n* = 107) and non-spironolactone (*n* = 427) groups is shown in Table 1. In the spironolactone group, it revealed lower eGFR (*p* < 0.001), and higher age (*p* = 0.02), furosemide prevalence (*p* < 0.001), furosemide daily dose (*p* < 0.001), s-K (*p* < 0.001), s-urea (*p* < 0.001), s-creatinine (*p* < 0.001), s-UA (*p* < 0.001), FE-K (*p* = 0.02), FE-Cl (*p* = 0.03), FE-H_2_O (*p* = 0.01), prevalences of hepatic disease (*p* < 0.001), chronic heart failure (*p* < 0.001), alcohol abuse (*p* = 0.045) and ascites (*p* < 0.001).

**Table 1.** Crude comparison of spironolactone versus non-spironolactone groups.

Admission parameters								
		Spironolactone (*n* = 107)			Non-spironolactone (*n* = 427)			
	Unit	Number	Median (IQR)	%	Number	Median (IQR)	%	*p* Value
Age	years	107	76 (62–85)		427	71 (59–81)		0.02
Female gender	–	66		61.7	259		60.7	0.92
Furosemide – prevalence	–	80		74.8	53		12.4	<0.001
Furosemide – daily dose	mg	78	40 (20–60)		52	20 (20–40)		<0.001
Spironolactone – daily dose	mg	102	25 (25–75)		–	–		–
*Serum*								
Na	mmol/L	107	119 (116–125)		427	122 (117–126)		0.08
K	mmol/L	98	4.7 (4–5.1)		389	4.1 (3.7–4.6)		<0.001
Cl	mmol/L	107	88 (84–93)		427	89 (84–93)		0.51
Osmolality (calculated)	mmol/kg	107	258 (245–266)		427	256 (246–264)		0.52
Osmolality (measured)	mmol/kg	56	249 (240–257)		210	247 (238–258)		0.59
Urea	mmol/L	107	7.2 (4.7–11.8)		427	4.1 (3–5.9)		<0.001
Creatinine	μmol/L	107	89 (67–126)		426	67 (53–91)		<0.001
eGFR (CKD-EPI)	mL/min/1.73 m^2^	107	59 (42–87)		426	84 (59–100)		<0.001
Uric acid	μmol/L	61	333 (258–486)		236	256 (180–345)		<0.001
Glucose	mmol/L	107	6.6 (5.8–7.9)		427	6.8 (5.9–8.3)		0.17
*Urine*								
Osmolality	mmol/kg	52	312 (211–371)		199	323 (206–465)		0.48
Na	mmol/L	56	39 (21–63)		195	40 (19–68)		0.92
K	mmol/L	56	27 (18–35)		195	28 (16–46)		0.37
Cl	mmol/L	56	40 (22–72)		195	39 (19–73)		0.76
*Fractional excretion*								
Na	%	45	1.15 (0.58–2.63)		145	0.91 (0.45–1.75)		0.08
K	%	52	13.9 (10.2–22.2)		187	12.4 (7.57–17.4)		0.02
Cl	%	45	1.65 (0.76–3.86)		146	1.18 (0.59–2.32)		0.03
Uric acid	%	35	10.8 (6.00–15.8)		127	10.7 (7.51–14.2)		0.93
H_2_O	%	56	2.67 (1.43–4.75)		195	1.96 (1.63–2.41)		0.01
*Comorbidities present on admission and in-hospital mortality*								
Hypertension	–	66		61.7	260		60.9	0.88
Hepatic disease/cirrhosis	–	57		53.3	131		30.7	<0.001
Chronic heart failure	–	45		42.1	41		9.6	<0.001
Alcohol abuse	–	36		33.6	103		24.1	0.045
Diabetes	–	36		33.6	108		25.3	0.08
Acute infection	–	30		28.0	141		33.0	0.32
GI losses	–	27		25.2	130		30.4	0.29
Chronic kidney disease	–	19		17.8	49		11.5	0.08
Ascites	–	19		17.8	12		2.8	<0.001
Active cancer	–	6		5.6	43		10.1	0.15
Mortality	–	16		15.0	40		9.4	0.09

IQR: interquartile range; eGFR: estimated glomerular filtration rate; GI: gastrointestinal.

Multivariable linear regression analyses performed for s-K, s-Na, FE-K, FE-Cl, FE-H_2_O and s-urea as dependent variables are shown in Table 2. In the multivariable linear regression model for s-K, lower eGFR (*b* = −0.01, *p* < 0.001) and spironolactone use (*b* = 0.33, *p* = 0.001) were independently associated with higher s-K. In the multivariable model for s-Na, alcohol abuse (*b* = −3.03, *p* < 0.001), GI (gastrointestinal) losses (*b* = −1.27, *p* = 0.03) and higher eGFR (*b* = −0.03, *p* = 0.006) were independently associated with lower s-Na. In the multivariable linear regression analyses for FE-K, FE-Cl and FE-H_2_O (following logarithmic transformation of dependent variables), lower eGFR was independently and inversely associated with FE-K (*b* = −0.01, *p* < 0.001; change = −0.7%), FE-Cl (*b* = −0.01, *p* < 0.001; change = −1.1%) and FE-H_2_O (*b* = −0.01, *p* = 0.001; change = −0.7%). In the multivariable model for s-urea (following logarithmic transformation of dependent variable), eGFR (*b* = −0.015, *p* < 0.001; change: −1.5%) and age (*b* = −0.01, *p* = 0.003; change: −0.5%) were independently associated with lower s-urea; spironolactone use (*b* = 0.22, *p* < 0.001; change: +24.5%) was independently associated with higher s-urea. No other variables were significant. The maximum VIF across all variables was 1.86.

**Table 2.** Multivariable linear regression analyses.

Dependent variable	*N*	*R* ^2^	Independent variable	Coefficient estimate *b*	95% CI for *b*	VIF	*p* Value	Change (%)^a^
s-K	487	0.135	eGFR	−0.008	−0.011 to −0.006	1.13	<0.001	–
			Furosemide use	−0.111	−0.291 to 0.069	1.56	0.23	–
			Spironolactone use	0.330	0.141 to 0.520	1.46	0.001	–
s-Na	533	0.086	Active cancer	0.463	−1.399 to 2.325	1.06	0.63	–
			Acute infection	0.142	−0.995 to 1.280	1.03	0.81	–
			Alcohol abuse	−3.025	−4.533 to −1.517	1.61	<0.001	–
			Ascites	−0.866	−3.373 to 1.640	1.27	0.50	–
			Furosemide use	−0.520	−2.166 to 1.126	1.87	0.54	–
			GI losses	−1.273	−2.433 to −0.114	1.02	0.03	–
			Hepatic disease/cirrhosis	0.217	−1.133 to 1.568	1.53	0.75	–
			eGFR	−0.031	−0.053 to −0.009	1.52	0.006	–
			Chronic heart failure	−0.982	−2.697 to 0.733	1.46	0.26	–
			Spironolactone use	−0.911	−2.591 to 0.769	1.67	0.29	–
			Age	−0.001	−0.049 to 0.048	1.69	0.98	–
s-urea^b^	533	0.559	Ascites	−0.131	−0.298 to 0.036	1.23	0.12	−12.3
			Furosemide use	0.017	−0.094 to 0.127	1.86	0.77	1.7
			Spironolactone use	0.219	0.106 to 0.332	1.64	<0.001	24.5
			Hepatic disease/cirrhosis	0.059	−0.023 to 0.141	1.24	0.16	6.1
			eGFR	−0.015	−0.017 to −0.014	1.51	<0.001	−1.5
			GI losses	−0.033	−0.111 to 0.045	1.02	0.41	−3.2
			Age	−0.005	−0.008 to −0.002	1.52	0.003	−0.5
			Chronic heart failure	0.077	−0.039 to 0.192	1.46	0.19	8.0
FE-K^b^	239	0.163	Spironolactone use	−0.020	−0.230 to 0.191	1.38	0.86	−2.0
			Furosemide use	0.207	−0.004 to 0.417	1.56	0.054	23.0
			eGFR	−0.007	−0.010 to −0.004	1.23	<0.001	−0.7
FE-Cl^b^	191	0.164	Furosemide use	0.344	−0.068 to 0.755	1.66	0.10	41.1
			Spironolactone use	0.106	−0.293 to 0.505	1.37	0.60	11.2
			eGFR	−0.011	−0.017 to −0.006	1.28	<0.001	−1.1
FE-H_2_O^b^	251	0.097	Furosemide use	0.225	−0.103 to 0.552	1.59	0.18	25.2
			Spironolactone use	0.117	−0.207 to 0.441	1.39	0.48	12.4
			eGFR	−0.007	−0.011 to −0.003	1.24	0.001	−0.7

VIF: variance inflation factor; eGFR: estimated glomerular filtration rate; GI: gastrointestinal.

^a^ Change (%) was calculated as (*e^b^* − 1) × 100.

^b^ These dependent variables were log-transformed to improve symmetry and stabilize variance.

A comparison of biochemical parameters adjusted for eGFR between the spironolactone (*n* = 107) and non-spironolactone (*n* = 426) groups is shown in Table 3. One patient in the non-spironolactone group was excluded because s-creatinine and, consequently, eGFR were unavailable. It established higher s-K (*p* < 0.001), s-urea (*p* < 0.001), s-UA (*p* = 0.01) and lower s-Na (*p* = 0.007) in the spironolactone group. No statistically significant difference was observed for the rest of the monitored parameters.

**Table 3.** Comparison of spironolactone versus non-spironolactone groups adjusted for eGFR (76.3 mL/min/1.73 m^2^).

Admission parameters								
		Spironolactone (*n* = 107)			Non-spironolactone (*n* = 426^a^)			
	Unit	Number	Median (IQR)	%	Number	Median (IQR)	%	*p* Value
*Serum*								
Na	mmol/L	107	119 (115–125)		426	122 (117–126)		0.007
K	mmol/L	98	4.5 (4.0–4.9)		389	4.2 (3.8–4.6)		<0.001
Cl	mmol/L	107	87 (83–93)		426	90 (85–93)		0.08
Osmolality (calculated)	mmol/kg	107	255 (245–264)		426	257 (248–264)		0.12
Osmolality (measured)	mmol/kg	56	248 (238–256)		210	248 (238–258)		0.60
Urea	mmol/L	107	6.0 (4.5–9.0)		426	5.3 (3.8–6.7)		<0.001
Creatinine	μmol/L	107	78 (62–93)		426	80 (65–98)		0.24
eGFR (CKD-EPI)	mL/min/1.73 m^2^	*n* = 533; mean = 76.3 ± 26.5 mL/min/1.73 m^2^						
Uric acid	μmol/L	61	322 (240–411)		235	264 (205–346)		0.01
Glucose	mmol/L	107	6.6 (5.8–7.9)		426	6.8 (5.9–8.3)		0.16
*Urine*								
Osmolality	mmol/kg	52	321 (227–373)		199	327 (210–462)		0.67
Na	mmol/L	56	39 (21–63)		195	40 (20–67)		0.83
K	mmol/L	56	26.7 (18.2–35.7)		195	28.2 (15.2–46.7)		0.51
Cl	mmol/L	56	40 (22–72)		195	39 (19–73)		0.53
*Fractional excretion*								
Na	%	45	1.10 (0.50–2.65)		145	1.10 (0.60–1.73)		0.80
K	%	52	14.6 (8.85–19.2)		187	13 (9.78–17.4)		0.97
Cl	%	45	1.30 (0.70–3.83)		146	1.5 (0.80–2.60)		0.82
Uric acid	%	35	10.8 (6.00–15.7)		127	10.7 (7.53–14.3)		0.89
H_2_O	%	56	2.00 (1.30–5.15)		195	2.10 (1.10–3.48)		0.40

IQR: interquartile range; eGFR: estimated glomerular filtration rate.

^a^ One patient in the non-spironolactone group was excluded because serum creatinine and, consequently, eGFR were unavailable.

To eliminate the effect of furosemide, we compared patients using spironolactone alone (*n* = 27) vs. those receiving neither spironolactone nor furosemide (*n* = 374; the group without diuretics). The results are shown in Table 4 and revealed higher age (*p* = 0.01), s-K (*p* = 0.003), s-urea (*p* = 0.001), prevalences of hypertension (*p* = 0.045), diabetes (*p* = 0.04) and ascites (*p* = 0.002) in the spironolactone alone group compared to the group with neither spironolactone nor furosemide.

**Table 4.** Comparison of spironolactone alone versus no diuretics groups.

Admission parameters											
			Spironolactone only (*n* = 27)			No diuretics (*n* = 374)					
	Unit		Number	Median (IQR)	%	Number	Median (IQR)		%		*p* Value
Age	years		27	80 (64–85)		374	70 (57–79)				0.01
Female gender	–		17		63.0	216			57.8		0.60
Spironolactone – dose	mg		26	25 (25–50)		–	–				–
*Serum*											
Na	mmol/L		27	119 (114–124)		374	122 (114–124)				0.06
K	mmol/L		25	4.5 (4.18–5.03)		337	4.1 (3.7–4.6)				0.003
Cl	mmol/L		27	87 (81–94)		374	89 (84–93)				0.69
Osmolality (calculated)	mmol/kg		27	253 (240–264)		374	256 (246–263)				0.27
Osmolality (measured)	mmol/kg		17	246 (239–257)		189	247 (238–258)				0.995
Urea	mmol/L		27	5.0 (4.4–9.1)		374	4.0 (2.9–5.6)				0.001
Creatinine	μmol/L		27	66 (52.8–82.8)		373	66 (53–87)				0.88
eGFR (CKD-EPI)	mL/min/1.73 m^2^		27	77 (62–92)		373	85 (63–102)				0.29
Uric acid	μmol/L		13	258 (194–312)		199	245 (171–328)				0.81
Glucose	mmol/L		27	6.3 (5.83–8.13)		374	6.7 (5.9–8.2)				0.50
*Urine*											
Osmolality	mmol/kg		15	318 (227–500)		172	326 (197–474)				0.91
Na	mmol/L		17	40 (21–67)		168	38 (19–66)				0.64
K	mmol/L		17	31 (17–41)		168	29 (15–48)				0.998
Cl	mmol/L		17	33 (22–76)		168	37 (19–68)				0.77
*Fractional excretion*											
Na	%		14	0.92 (0.44–2.31)		123	0.81 (0.42–1.69)				0.47
K	%		16	11.0 (8.82–15.7)		161	11.7 (7.30–15.9)				0.57
Cl	%		13	1.12 (0.67–3.48)		123	1.09 (0.51–2.08)				0.40
Uric acid	%		10	12.7 (7.9–18.3)		106	10.5 (7.28–14.0)				0.29
H_2_O	%		17	2.06 (1.07–4.44)		168	1.86 (0.86–3.2)				0.29
*Comorbidities present on admission and in-hospital mortality*											
Hypertension		–	21		77.8	216		57.8		0.045	
Hepatic disease/cirrhosis		–	13		48.1	122		32.6		0.10	
Chronic heart failure		–	3		11.1	19		5.1		0.19	
Alcohol abuse		–	8		29.6	99		26.5		0.73	
Diabetes		–	11		40.7	87		23.3		0.04	
Acute infection		–	9		33.3	118		31.6		0.86	
GI losses		–	10		37.0	118		31.6		0.56	
Chronic kidney disease		–	4		14.8	39		10.4		0.48	
Ascites		–	4		14.8	11		2.9		0.002	
Active cancer		–	2		7.4	36		9.6		0.71	
Mortality		–	2		7.4	33		8.8		0.80	

IQR: interquartile range; eGFR: estimated glomerular filtration rate; GI: gastrointestinal.

Correlations are presented in Table 5. We evaluated the correlation between spironolactone dose and biochemical parameters. To account for the influence of furosemide, the spironolactone dose was adjusted for the furosemide dose. We observed a negative correlation between spironolactone dose and u-Na and u-Cl, both in unadjusted analyses (*r*_s_ = −0.43, *p* = 0.002; *r*_s_ = −0.32, *p* = 0.02, respectively) and after adjustment for furosemide dose (*r*_s_ = −0.36, *p* = 0.01; *r*_s_ = −0.28, *p* = 0.04, respectively). After adjustment for furosemide dose, we observed a negative correlation between spironolactone dose and (i) FE-Cl (*r*_s_ = −0.33, *p* = 0.04) and (ii) FE-H_2_O (*r*_s_ = −0.29, *p* = 0.03).

**Table 5.** Correlations between spironolactone dose and biochemical parameters.

	Spironolactone dose (*n* = 102)					
	Unadjusted			Adjusted for furosemide dose		
	*N*	*R*(*s*)	*p* Value	*N*	*R*(*s*)	*p* Value
*Serum*						
Na	102	−0.11	0.27	102	−0.11	0.27
K	94	−0.03	0.81	94	−0.01	0.95
Cl	102	−0.01	0.96	102	0.01	0.94
Osmolality (calculated)	102	−0.15	0.14	102	−0.16	0.11
Osmolality (measured)	55	0.11	0.44	55	0.10	0.48
Urea	102	−0.08	0.42	102	−0.15	0.13
Creatinine	102	−0.04	0.67	102	−0.08	0.42
eGFR (CKD-EPI)	102	0.16	0.11	102	0.19	0.06
Uric acid	59	0.14	0.28	59	0.12	0.36
Glucose	102	−0.03	0.80	102	−0.02	0.84
*Urine*						
Osmolality	49	−0.01	0.92	49	0.02	0.90
Na	53	−0.43	0.002	53	−0.36	0.01
K	53	0.03	0.81	53	0.09	0.50
Cl	53	−0.32	0.02	53	−0.28	0.04
*FE*						
Na	42	−0.14	0.39	42	−0.24	0.12
K	50	−0.14	0.33	50	−0.17	0.23
Cl	42	−0.23	0.15	42	−0.33	0.04
Uric acid	35	−0.14	0.44	35	−0.09	0.62
H_2_O	53	−0.15	0.28	53	−0.29	0.03
*s-K* (*n* = 94)						
	*N*		*R*(*s*)		*p* Value	
u-K	50		0.09		0.53	
FE-K	50		0.18		0.23	

*R*(*s*): Spearman’s correlation coefficient; eGFR: estimated glomerular filtration rate.

To clarify the extent to which furosemide affects the biochemical profile when combined with spironolactone, we compared biochemical and clinical characteristics of patients taking spironolactone alone (*n* = 27) vs. patients using spironolactone together with furosemide (*n* = 80). The results are presented in Table 6. They show lower spironolactone dose (*p* = 0.04), s-urea (*p* = 0.04), s-creatinine (*p* < 0.001), s-UA (*p* = 0.02) and prevalence of chronic heart failure (*p* < 0.001); higher eGFR (*p* = 0.002) and prevalence of hypertension (*p* = 0.048) in the spironolactone-alone group in comparison to the group using spironolactone together with furosemide.

**Table 6.** Comparison of spironolactone alone versus spironolactone + furosemide groups.

Admission parameters								
		Spironolactone alone (*n* = 27)			Spironolactone + furosemide (*n* = 80)			
	Unit	Number	Median (IQR)	%	Number	Median (IQR)	%	*p* Value
Age	years	27	80 (64–85)		80	74 (62–86)		0.42
Female gender	–	17		63.0	49		61.3	0.89
Spironolactone – dose	mg	26	25 (25–50)		76	37.5 (25–75)		0.04
Furosemide – dose	mg	–	–		78	40 (20–60)		–
*Serum*								
Na	mmol/L	27	119 (114–124)		80	120 (117–125)		0.23
K	mmol/L	25	4.5 (4.18–5.03)		73	4.7 (3.98–5.13)		0.997
Cl	mmol/L	27	87 (81–94)		80	88 (84–93)		0.86
Osmolality (calculated)	mmol/kg	27	253 (240–264)		80	260 (247–267)		0.08
Osmolality (measured)	mmol/kg	17	246 (239–257)		39	250 (241–257)		0.62
Urea	mmol/L	27	5.0 (4.4–9.1)		80	7.5 (5.4–12.4)		0.04
Creatinine	μmol/L	27	66 (52.8–82.8)		80	98.5 (77–129)		<0.001
eGFR (CKD-EPI)	mL/min/1.73 m^2^	27	77 (62–92)		80	54 (38–73)		0.002
Uric acid	μmol/L	13	258 (194–312)		48	363 (296–489)		0.02
Glucose	mmol/L	27	6.3 (5.83–8.13)		80	6.7 (5.6–7.9)		0.88
*Urine*								
Osmolality	mmol/kg	15	318 (227–500)		37	312 (208–359)		0.46
Na	mmol/L	17	40 (21–67)		39	38 (20–58)		0.62
K	mmol/L	17	31 (17–41)		39	23 (18–33)		0.43
Cl	mmol/L	17	33 (22–76)		39	41 (22–70)		0.89
*Fractional excretion*								
Na	%	14	0.92 (0.44–2.31)		31	1.29 (0.694–3.08)		0.33
K	%	16	11.0 (8.82–15.7)		36	15.7 (11.7–23.2)		0.08
Cl	%	13	1.12 (0.67–3.48)		32	1.89 (0.78–4.83)		0.23
Uric acid	%	10	12.7 (7.9–18.3)		25	10.3 (6.0–13.4)		0.24
H_2_O	%	17	2.06 (1.07–4.44)		39	2.8 (1.54–5.61)		0.24
*Comorbidities present on admission and in-hospital mortality*								
Hypertension	–	21		77.8	45		56.3	0.048
Hepatic disease/cirrhosis	–	13		48.1	44		55.0	0.53
Chronic heart failure	–	3		11.1	42		52.5	<0.001
Alcohol abuse	–	8		29.6	28		35.0	0.61
Diabetes	–	11		40.7	25		31.3	0.37
Acute infection	–	9		33.3	21		26.3	0.49
GI losses	–	10		37.0	17		21.3	0.11
Chronic kidney disease	–	4		14.8	15		18.8	0.64
Ascites	–	4		14.8	15		18.8	0.64
Active cancer	–	2		7.4	4		5.0	0.64
Mortality	–	2		7.4	14		17.5	0.21

IQR: interquartile range; eGFR: estimated glomerular filtration rate; GI: gastrointestinal.

## 4. Discussion

Spironolactone was independently associated with higher s-K and with higher s-urea levels. This pattern was not reflected by reduced urinary potassium excretion. Importantly, no clear evidence that concomitant furosemide materially modified this biochemical pattern was observed in the present analyses.

The prevalence of spironolactone among patients in the non-thiazide arm was 20.0% (107/534). In comparison, the prevalence of spironolactone in the thiazide arm reached 6.2% (18/290) [18]. Notably, 74.8% (80/107) of patients in the non-thiazide arm used spironolactone with concomitant furosemide therapy. This may simply reflect its general use pattern as indications for treatment with spironolactone (e.g. liver cirrhosis-associated ascites and heart failure) are often associated with the need to add a loop diuretic [19,20]. However, the combination of spironolactone with furosemide is a well-known risk factor for the development of hypotonic hyponatraemia in patients with heart failure [15].

Patients receiving spironolactone were older (*p* = 0.02) and exhibited worse renal functions, defined as higher s-urea (*p* < 0.001), s-creatinine (*p* < 0.001), s-UA (*p* < 0.001) and lower eGFR (*p* < 0.001) compared to patients in the non-spironolactone group (Table 1). The higher age and impaired renal function can be associated with comorbidities for which spironolactone is typically prescribed. Although the absolute median eGFR value of 59 mL/min [IQR: 42–87] in the spironolactone group was not low enough to expect the major biochemical disturbances typically seen in chronic kidney disease [21,22], it remains appropriate to take into consideration the significantly lower eGFR in the spironolactone group compared to the non-spironolactone group.

The increased FE-K (*p* = 0.02), FE-Cl (*p* = 0.03) and FE-H_2_O (*p* = 0.01) observed in the spironolactone group compared to the non-spironolactone group may be attributed to the effect of furosemide [23] and/or a compensatory increase in FE in patients with chronic kidney disease [21]. Also, the elevated s-K (*p* < 0.001) observed in the spironolactone group, in comparison with the non-spironolactone group, may reflect lower renal function [22] and/or the pharmacological effect of spironolactone [9,24,25].

To further explore the relationship between s-K and potential determinants, multivariable linear regression analysis was performed, demonstrating both lower eGFR (*b* = −0.01, *p* < 0.001) and spironolactone use (*b* = 0.33, *p* = 0.001) were independently associated with higher s-K levels (Table 2). This finding supports an adjusted association between spironolactone use and higher s-K within this cohort of patients with established hypotonic hyponatraemia. In multivariable linear regression analyses with log-transformed dependent variables, eGFR was also independently and inversely associated with FE-K (*b* = −0.01, *p* < 0.001; change = −0.7%), FE-Cl (*b* = −0.01, *p* < 0.001; change = −1.1%) and FE-H_2_O (*b* = −0.01, *p* = 0.001; change = −0.7%).

Therefore, comparison of biochemical parameters adjusted for eGFR was performed, revealing higher s-K (*p* < 0.001) and lower s-Na (*p* = 0.007) in the spironolactone group compared with the non-spironolactone group (Table 3). However, in the fully adjusted multivariable model, spironolactone use was not independently associated with s-Na (*b* = −0.91, *p* = 0.29; Table 2). In this model, alcohol abuse (*b* = −3.03, *p* < 0.001), GI losses (*b* = −1.27, *p* = 0.03) and higher eGFR (*b* = −0.03, *p* = 0.006) were independently associated with lower s-Na.

Notably, the higher prevalence of furosemide use in the spironolactone group (74.8%; 80/107) compared to the non-spironolactone group (12.4%; 53/427), along with a higher median daily dose (40 mg [IQR: 20–60] vs. 20 mg [IQR: 20–40], respectively), did not counteract the association between spironolactone use and higher s-K (Table 1). Consistent with this finding, multivariable linear regression analysis did not demonstrate an independent association between furosemide use (*b* = −0.11, *p* = 0.23) and s-K (Table 2). Moreover, no clear evidence that co-administration of furosemide materially modified FE was observed in the present analyses, as the differences in FE remained statistically non-significant after adjusting for eGFR (Table 3) and multivariable linear regression identified no significant association between furosemide use and FE (Table 2). For reference, in a study involving patients with cirrhosis and ascites who were treated with a combination of furosemide and spironolactone, spironolactone was associated with a higher risk of hyponatraemia regardless of the furosemide dose [16]. The lack of clear evidence that furosemide materially modified the biochemical pattern of patients using spironolactone may hypothetically be related to furosemide resistance [26]. However, furosemide resistance was not assessed in the present study, and its potential role in these findings cannot be determined.

The association between spironolactone use and higher s-K (*p* = 0.003) was observed when comparing the group treated with spironolactone alone to the group without diuretics (Table 4). Notably, the higher s-K associated with spironolactone use was not accompanied by lower u-K and/or FE-K after adjustment for eGFR (Table 3) or after excluding the effect of furosemide (Table 4). Furthermore, we did not detect a correlation between s-K and u-K (*r*_s_ = 0.09, *p* = 0.53) and/or FE-K (*r*_s_ = 0.18, *p* = 0.23) in the spironolactone group (Table 5). The plausible explanation for the observed hyperkalaemia associated with spironolactone use, which was not accompanied by renal potassium retention, may be long-term use of spironolactone. This may result in the establishment of a new steady-state of s-K, with normalization of renal potassium excretion to baseline levels [27].

Another reason may be a compensatory increase in aldosterone activity at the time of admission. Spironolactone use was associated with higher s-urea in the crude comparison (*p* < 0.001; Table 1), and more importantly, in conditions without differences in eGFR and s-creatinine: after adjustment for eGFR (*p* < 0.001; Table 3), and after filtering out the effect of furosemide (*p* = 0.001; Table 4). An increase in s-urea could be interpreted as a marker of reduced EABV [28,29]. A reduction in EABV naturally activates the renin–angiotensin–aldosterone system [30,31] and counteracts the pharmacological effect of spironolactone [32], particularly given the relatively low dose administered in our study (median daily dose 25 mg [IQR: 25–75]). Further, spironolactone use was independently associated with higher s-urea in the multivariable linear regression analysis (*b* = 0.22, *p* < 0.001). This coefficient corresponds to an average increase in s-urea of 24.5% (95% CI: 11.2–39.3%) in patients treated with spironolactone compared to those untreated, even after adjusting for age, eGFR, chronic heart failure, liver disease and other clinical factors (Table 2). Therefore, the association between spironolactone use and higher s-urea may be compatible with differences in volume status, but s-urea is nonspecific and the underlying mechanism cannot be determined from the present data. Regardless of why the spironolactone-associated rise in s-K was not mirrored by reduced renal potassium excretion, within this cohort, urinary potassium and fractional potassium excretion did not clearly distinguish hyponatraemic patients receiving spironolactone from comparison patients.

The positive correlation between spironolactone dose and s-K is well described [9,24,25], but in certain populations, such as gender-diverse adolescents [33] or patients with HFpEF and concomitant CKD [34], this relationship may not be straightforward. In addition, in specific pathophysiological states, including heart failure or ascites, the therapeutic effect of spironolactone may only become apparent at higher doses [32]. In our cohort, we found no correlation between spironolactone dose and s-K (*r*_s_ = −0.026, *p* = 0.81; Table 5). This lack of correlation observed in the current study may be explained by the nonlinear and interindividual variability of its effect at low to moderate doses, particularly under conditions of activated RAAS.

It did not escape our attention that removing the effect of furosemide led to the disappearance of the statistically significant difference in eGFR between the group treated with spironolactone alone and the group without diuretics (Table 4). In accordance with this finding, comparison of groups treated with spironolactone alone vs. spironolactone together with furosemide has revealed an association of furosemide with lower eGFR (Table 6). This is not surprising and likely reflects the need to increase fluid excretion in patients with higher prevalence of chronic heart failure (*p* < 0.001). The higher spironolactone dose in patients receiving concomitant furosemide, compared with those on spironolactone alone (*p* = 0.04), aligns with a previous study in patients with heart failure and hyponatraemia [15].

### 4.1. Strengths and limitations

This study provides detailed biochemical profiling with eGFR adjustment and multivariable linear regression analyses, allowing a detailed description of biochemical patterns associated with spironolactone use. Moreover, its real-world retrospective design reflects routine clinical practice, including frequent co-administration with furosemide. Although single-centre, the observed prescribing patterns and patient characteristics reflect routine internal medicine practice, supporting the clinical relevance of the findings.

On the other hand, these findings must be interpreted in consideration of several limitations. First, due to the retrospective design, potential sources of bias include incomplete medication histories, inability to verify adherence or timing of last diuretic doses, and residual confounding related to comorbidities influencing diuretic prescription. In particular, s-urea is a nonspecific marker potentially influenced by other factors that we were unable to include in the analyses. Second, some measurements are incomplete. Third, we were unable to determine the volemic status of patients. Fourth, this study was exploratory in nature, and no *a priori* sample size calculation was performed. The sample size was determined by the number of eligible patients admitted during the study period. Fifth, subgroup analyses, particularly those excluding concomitant furosemide use, were limited by relatively small group sizes and should therefore be interpreted with caution. Sixth, for determining eGFR, it would be more appropriate to also use cystatin C [35]. Seventh, in the multivariable models, spironolactone and furosemide were analysed as binary exposure variables rather than according to prescribed daily dose. Although medication dose was examined in separate analyses, potential dose-dependent effects may not have been fully captured.

## 5. Conclusions

In this real-world cohort of hospitalized patients with hypotonic hyponatraemia, spironolactone use was associated with a distinct biochemical pattern characterized by independent associations with higher s-K and higher s-urea levels. This pattern was not reflected by reduced urinary potassium excretion. Importantly, no clear evidence that concomitant furosemide materially modified this biochemical pattern was observed in the present analyses. Awareness of this pattern may support the clinical assessment and phenotypic characterization of patients with hyponatraemia receiving spironolactone. However, because the observational design does not permit causal inference, prospective studies are warranted to evaluate the diagnostic utility of this biochemical pattern in hyponatraemic patients receiving spironolactone.

## Supplementary Material

Spironolactone_raw_data.xlsx

## Data Availability

The anonymized data supporting the findings of this study are available in the supplementary materials.
